# A226 NEUTROPHIL-TO-LYMPHOCYTE RATIO AT DIAGNOSIS PREDICTS COLONOSCOPIC ACTIVITY IN PEDIATRIC INFLAMMATORY BOWEL DISEASES (PIBD)

**DOI:** 10.1093/jcag/gwad061.226

**Published:** 2024-02-14

**Authors:** B Aziz, R Belaghi, H Huynh, K Jacobson, D Mack, C Deslandres, A Otley, J deBruyn, W El-Matary, E Crowley, M Sherlock, N Ahmed, A Griffiths, T Walters, E Wine

**Affiliations:** Pediatrics, University of Alberta, Edmonton, AB, Canada; Children's Hospital of Eastern Ontario, Ottawa, ON, Canada; Pediatrics, University of Alberta, Edmonton, AB, Canada; The University of British Columbia, Vancouver, BC, Canada; University of Ottawa, Ottawa, ON, Canada; Universite de Montreal, Montreal, QC, Canada; Dalhousie University, Halifax, NS, Canada; University of Calgary, Calgary, AB, Canada; University of Manitoba, Winnipeg, MB, Canada; Western University, London, ON, Canada; McMaster University, Hamilton, ON, Canada; Montreal Children's Hospital, Montreal, QC, Canada; University of Toronto, Toronto, ON, Canada; University of Toronto, Toronto, ON, Canada; Pediatrics, University of Alberta, Edmonton, AB, Canada

## Abstract

**Background:**

Neutrophil-to-lymphocyte ratio (NLR) has been recently identified as a potential biomarker for several autoimmune conditions. NLR predicts disease activity in adults with inflammatory bowel diseases (IBD) but had not been studied in pediatric IBD.

**Aims:**

Investigate the link between NLR and baseline colonoscopic disease activity in UC and CD, the need for surgery or admission, and one-year therapy response in children.

**Methods:**

Pediatric IBD patients were prospectively enrolled into the CIDsCaNN database [Canada-wide inception cohort for pediatric patients with IBD]. The cohort included patients diagnosed between 2003–2022 based on ileocolonoscopy, histopathology, and established diagnostic criteria. Patients were excluded if they had other conditions affecting NLR, such as neoplasms and autoimmune diseases. Endoscopic disease activity was assessed using the Mayo endoscopic score (MES; dichotomized as low (0, 1) or high activity (2, 3)) in UC and SES-CD score (as continuous variable) in CD. Logistic regression was used to test the link between predictors and binary outcomes. Simple linear regression was used to analyze the relationship between continuous variables.

**Results:**

576 UC and 1076 CD patients ampersand:003C18 years old were included. In UC, baseline NLR was significantly associated with baseline MES in univariate and multivariable logistic regression (multivariable OR= 1.5, 95% CI= 1.1–1.99, p= 0.019; Table). Patients with high-activity MES have significantly higher neutrophils (p= 0.0003), but lymphocytes wasn't different between the two groups. In CD, NLR was significantly correlated with SES-CD score in univariate and multivariable simple linear regression (multivariable coefficient= 1.4, 95% CI= 0.7–2.2, pampersand:003C0.0001; Fig1). Additionally, NLR predicted biologic-free remission in the CD cohort only in univariate analysis. NLR did not predict other the need for admission or surgery either in UC or CD.

**Conclusions:**

Pediatric IBD patients with higher baseline NLR had a higher endoscopic disease activity. This establishes the significance of NLR as a non-invasive biomarker to help direct clinical decision making. This association suggests a role for neutrophils, as innate immunity cells in the acute stage of inflammation.

Multivariable logistic regression of the relation between baseline labs in pediatric UC and Mayo endoscopic score

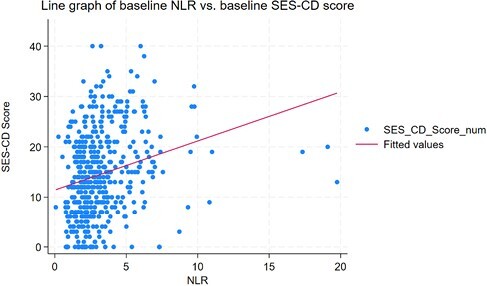

Scatter plot demonstrating the relationship between baseline NLR and baseline SES-CD score

**Funding Agencies:**

C.H.I.L.D.

**Table:** 

Variables	OR	Standard error	p value	95% CI
NLR	1.45	0.23	0.02	1.06 - 1.99
Albumin	0.91	0.03	0.002	0.85 - 0.96
Year of diagnosis	1.10	0.14	0.48	0.85 - 1.41
Extra intestinal manifestations	0.61	0.28	0.28	0.25 - 1.49

